# Skin Doctor: Machine Learning Models for Skin Sensitization Prediction that Provide Estimates and Indicators of Prediction Reliability

**DOI:** 10.3390/ijms20194833

**Published:** 2019-09-28

**Authors:** Anke Wilm, Conrad Stork, Christoph Bauer, Andreas Schepky, Jochen Kühnl, Johannes Kirchmair

**Affiliations:** 1 Center for Bioinformatics, Universität Hamburg, 20146 Hamburg, Germany; wilm@zbh.uni-hamburg.de (A.W.); stork@zbh.uni-hamburg.de (C.S.); 2 HITeC e.V, 22527 Hamburg, Germany; 3 Department of Chemistry, University of Bergen, 5020 Bergen, Norway; christoph.bauer@uib.no; 4 Computational Biology Unit (CBU), University of Bergen, 5020 Bergen, Norway; 5 Front End Innovation, Beiersdorf AG, 20253 Hamburg, Germany; andreas.schepky@beiersdorf.com (A.S.); jochen.kuehnl@beiersdorf.com (J.K.)

**Keywords:** skin sensitization potential, prediction, in silico models, machine learning, local lymph node assay (LLNA), cosmetics, drugs, pesticides, chemical space, applicability domain

## Abstract

The ability to predict the skin sensitization potential of small organic molecules is of high importance to the development and safe application of cosmetics, drugs and pesticides. One of the most widely accepted methods for predicting this hazard is the local lymph node assay (LLNA). The goal of this work was to develop in silico models for the prediction of the skin sensitization potential of small molecules that go beyond the state of the art, with larger LLNA data sets and, most importantly, a robust and intuitive definition of the applicability domain, paired with additional indicators of the reliability of predictions. We explored a large variety of molecular descriptors and fingerprints in combination with random forest and support vector machine classifiers. The most suitable models were tested on holdout data, on which they yielded competitive performance (Matthews correlation coefficients up to 0.52; accuracies up to 0.76; areas under the receiver operating characteristic curves up to 0.83). The most favorable models are available via a public web service that, in addition to predictions, provides assessments of the applicability domain and indicators of the reliability of the individual predictions.

## 1. Introduction

Repeated exposure to reactive chemicals with skin-sensitizing properties can cause allergic contact dermatitis (ACD) [1], an adverse cutaneous condition with a prevalence of ~20% among the general population [2] and even higher prevalence among workers with chronic occupational exposure [3]. Understanding the skin sensitization potential of small organic molecules is therefore of essence to the development and safe application of chemicals, including cosmetics and drugs.

Historically, animal tests have effectively been the only method for determining the skin sensitization potential and potency of substances. The local lymph node assay (LLNA) is currently considered to be the most advanced animal testing system [4]. In recent years, ethical considerations and regulatory requirements have led to an intensification of the search for alternatives to animal testing, in particular in the cosmetics industry [5]. New in vitro and in chemico methods have been developed and evaluated [6,7,8,9], and computational approaches are starting to be recognized as important alternatives to animal testing [8,9,10,11]. The non-redundant combinatorial use of said methods in defined approaches that assess several key events of the adverse outcome pathway (AOP) for skin sensitization shows promising predictive capacity [12] and is currently evaluated in risk assessment case studies.

The bottleneck in the development of in silico tools for the prediction of skin sensitization is not related to technology but to the scarcity of available high-quality experimental data for model development. Three strategies have been pursued to address this problem. The first one is to increase the amount and coverage of data by employing data mining techniques to retrieve information from various types of assays and sources [13,14]. Although this has been discussed as a promising strategy to increase the applicability of models, it has also prompted controversial discussions regarding the quality and relevance of the data [15,16]. The second strategy is to develop focused models based on small, focused data sets of high-quality [17,18,19,20,21]. The third strategy is to pursue a middle way that aims for a favorable balance between quantity and quality of the data. The LLNA data available in the public domain are generally regarded as the most suitable source of information for this strategy [22,23,24,25,26,27].

The two largest curated collections of LLNA outcomes in the public domain are the data collections of Alves et al. [28] and Di et al. [22]. The data were obtained from reliable sources and subjected to deduplication procedures that reject discordant records. The data set of Alves et al. includes (mainly) binary LLNA outcomes recorded for 1000 compounds. In addition, it contains human data and outcomes from different types of in vitro and in chemico assays, although for substantially fewer substances. Based on these data, the authors developed machine learning models for different assay types and also a consensus model, all of which are available via an online platform (“PredSkin”) [19]. Their model for the prediction of binary LLNA outcomes reached a correct classification rate (CCR) of 0.77 during five-fold external cross-validation.

The data set published by Di et al. contains 1007 substances annotated with LLNA potency classes [22]. Based on a subset of approximately 400 compounds for which an explicit reaction mechanism could be derived with a structural alerts tool for protein binding implemented in the OECD Toolbox [29], Di et al. developed a variety of models for the binary and ternary prediction of the skin sensitization potential. These models included local models for four reaction domains as well as global models. The best binary global model was reported to obtain an accuracy (ACC) of 0.84 during cross-validation and an ACC of 0.81 on a test set.

Major challenges in the application of machine learning approaches for risk assessment are related to the complexity of models that goes along with limited mechanistic interpretability. For these types of models, transparency with respect to the applicability domain as well as the provision of confidence estimates for individual predictions are of utmost importance to risk assessors, who ultimately are the main stakeholders of these methods.

In this context, and building on the works of Alves et al. and Di et al., this study pursues four main objectives to advance in silico capabilities for the prediction of the skin sensitization potential: (i) the development of a detailed understanding of the chemical space covered by the available LLNA data with respect to the chemical space of cosmetics, approved drugs and pesticides, (ii) the identification of the most suitable (sets of) molecular descriptors for modeling, (iii) the maximization of the applicability of the models by increasing the size and coverage of the data set used for model development, (iv) the definition of robust measures of the models’ applicability domain as well as the provision of indicators for the reliability of individual predictions, and (v) the provision of the most suitable models via a public web service.

## 2. Results

### 2.1. Characterization of the LLNA Data Sets

In order to develop a detailed understanding of the relevance of the available LLNA data to modeling the skin sensitization potential of xenobiotics, we analyzed the composition and molecular diversity of the LLNA data sets of Alves et al. and Di et al. In addition, we assessed how well the individual LLNA data sets cover the chemical space of cosmetics, approved drugs and pesticides.

#### 2.1.1. Data Set Composition

Whereas the data set compiled by Alves et al. is balanced (481 sensitizers; 519 non-sensitizers), the data set of Di et al. contains almost twice as many non-sensitizers (*n* = 629) as sensitizers (*n* = 364; Table 1). Roughly 40% of all compounds (567) are present in both data sets (Table 2). The LLNA data set compiled by Alves et al. contains 7% of all substances listed in the cosmetics data set; coverage is lower for approved drugs and pesticides (4% and 5%, respectively). The percentages are similar for the LLNA data set of Di et al.: 5% overlaps with cosmetics, 3% with approved drugs and 4% with pesticides. Merging the two LLNA data sets increases the number of unique compounds to 1416 and the overlaps with cosmetics, approved drugs and pesticides to 8%, 5% and 5%, respectively.

#### 2.1.2. Coverage of Chemical Space

**Figure 1:**
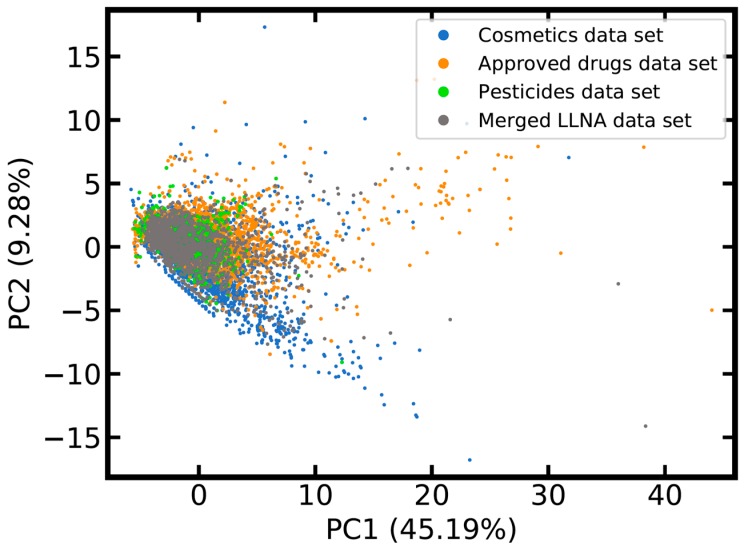
Score plot comparing the chemical space of compounds of the merged LLNA data set, cosmetics, approved drugs and pesticides. The plot is derived from a principal component analysis (PCA) based on 53 intuitive and physically meaningful molecular descriptors such as molecular weight and clogP (see Methods and Table S1 for details). Data points located in the lower parts of the PCA score plot are primarily cosmetics with long aliphatic and often halogenated chains; towards the top right corner of the diagram these are primarily large drug molecules with strong aromatic components. The variance explained by the first two principal components is reported in the axis titles. Four compounds of the cosmetics reference set and eight compounds of the approved drugs reference set are not shown because they are off the chosen limits of the plot (these are complex and large molecules, with a molecular weight of 2800 Da and higher).

Whereas only few of the cosmetics, approved drugs and pesticides listed in the reference data sets are included in the LLNA data sets, principal component analysis (PCA) shows that the areas in chemical space most densely populated with these xenobiotics are actually well-covered by the merged LLNA data set (Figure 1). Nevertheless, scattered data points radiating from the area of high data density towards the bottom and the top right corner of the PCA score plot indicate the existence of drugs and cosmetic compounds without closely related substances listed in the merged data set.

**Figure 2:**
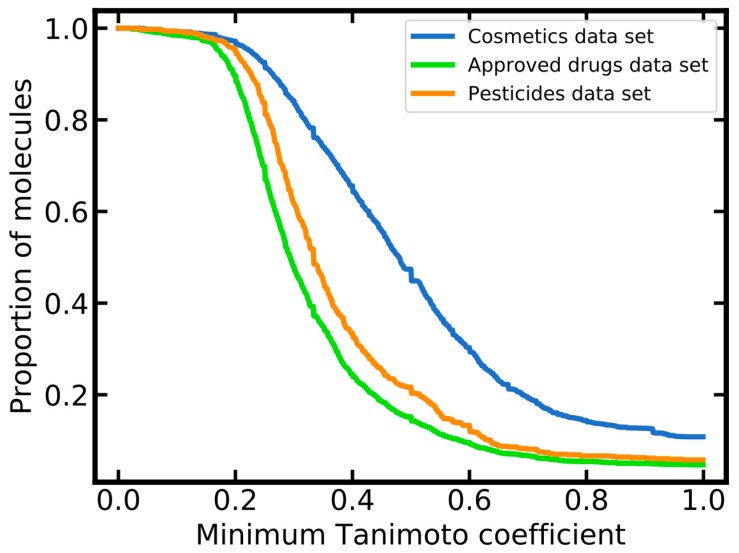
Molecular similarity between each compound of the reference data sets (i.e., cosmetics, approved drugs and pesticides data sets) and its nearest neighbor in the merged local lymph node assay (LLNA) data set (similarity quantified as Tanimoto coefficient based on Morgan2 fingerprints with a length of 2048 bits).

In addition to PCA analysis, the coverage of cosmetics, approved drugs and pesticides by the merged LLNA data set was quantified based on the distribution of maximum pairwise similarities. As shown in Figure 2, the merged LLNA data set covers cosmetics much better than approved drugs and pesticides: over 30% of all cosmetics are represented by the respective nearest neighbor in the merged LLNA data set with a minimum Tanimoto coefficient of 0.6, whereas this is the case for only 10% and 13% of all approved drugs and pesticides, respectively.

**Figure 3:**
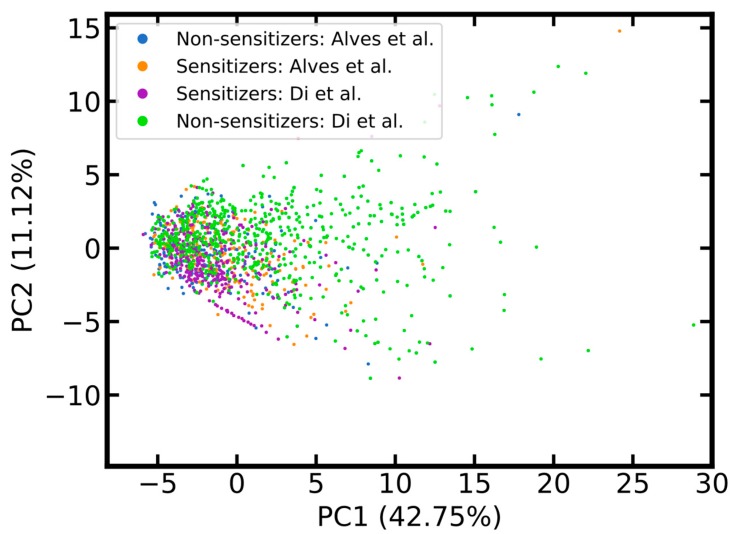
Score plot comparing the chemical space of compounds of the local lymph node assay (LLNA) data sets of Alves et al. and Di et al. The score plot was derived from a PCA based on the identical setup described in the caption of Figure 1. Two data points are located outside the displayed intervals.

It is important to note that the data set compiled by Di et al. includes many compounds that populate areas in chemical space not (well) covered by the LLNA data set of Alves et al. (Figure 3). It is therefore expected that models trained on the merged data set should be more widely applicable than those based solely on the LLNA data compiled by Alves et al.

#### 2.1.3. Molecular Diversity

**Figure 4:**
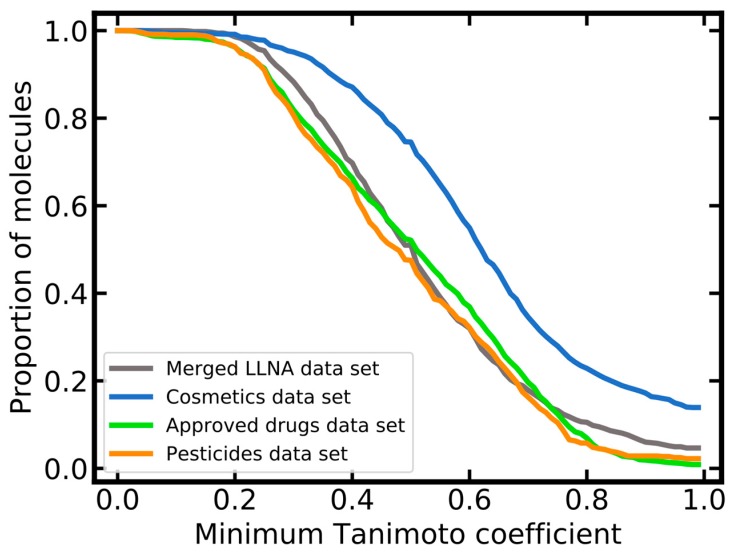
Pairwise molecular similarity within the individual data sets (similarity quantified as Tanimoto coefficient based on Morgan2 fingerprints with a length of 2048 bits).

The molecular diversity of the merged LLNA data set and the reference data sets was assessed in two different ways: by pairwise comparison of molecular structures and by counting of Murcko scaffolds. Pairwise comparisons were again based on Tanimoto coefficients derived from Morgan2 fingerprints of a length of 2048 bits. The cosmetics data set exhibits a lower diversity compared to the other data sets (Figure 4). This can be attributed, to some extent, to the larger size of the cosmetics data set: 23% of all pairs of compounds in the cosmetic data set have fingerprints with a Tanimoto coefficient of 0.8 or higher, whereas this percentage is 11% or lower for the merged LLNA, approved drugs and pesticides data sets. Of all compounds included in the cosmetics data set, 220 have at least one neighbor with identical molecular fingerprint. These are mostly pairs of molecules with long aliphatic chains, differing only by the length of these chains (note that any duplicate molecules have been removed during data preprocessing).

The merged LLNA data set covers a total of 453 distinct Murcko scaffolds, which is roughly as many as covered by the pesticides data set but only one-third and one-quarter of those covered by the cosmetics and approved drugs data sets, respectively (Table 1). Taking into account the size of the individual data sets, the approved drugs data set clearly is the most diverse data set. In contrast, the cosmetics data set, which counts more molecular structures than all other data sets taken together, is the least diverse data set. This is in part related to the fact that approximately 40% of all cosmetics do not include a ring and, as such, do not have a Murcko scaffold.

Benzene is the most prominent Murcko scaffold across all data sets, with a prevalence of 27%, 28%, 10% and 23% among the merged LLNA, cosmetics, approved drugs and pesticides data sets. Any other scaffolds are represented by only a few instances (Table S2). Note the high percentages of singleton scaffolds (72% or higher) across all data sets, which, particularly in the case of the LLNA data set, illustrate the scarcity of the data available for modeling.

### 2.2. Molecular Properties of Skin Sensitizers and Non-Sensitizers

**Figure 5:**
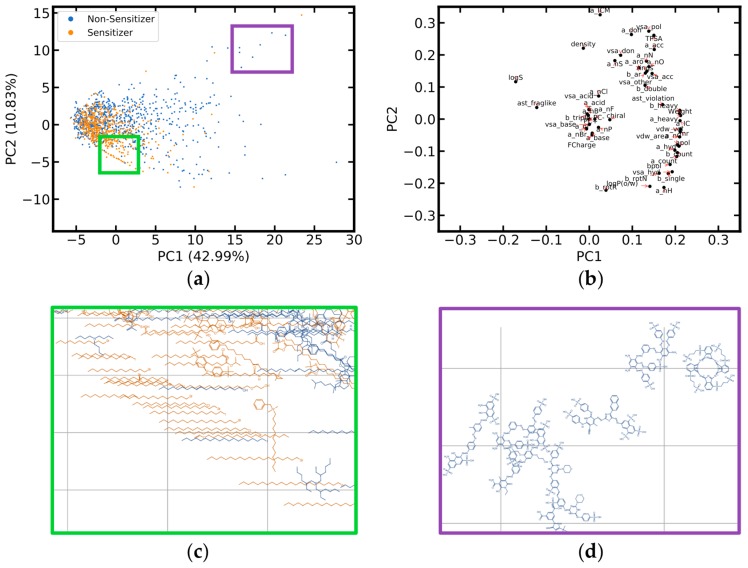
Principal component analysis (PCA) of the physicochemical properties of skin sensitizers and non-sensitizers included in the merged local lymph node assay (LLNA) data set. The PCA is based on the identical setup described in the caption of Figure 1. (**a**) Score plot, with the percentage of variance explained by the individual principal components reported as part of the axis labels. Two data points are located outside the displayed intervals. (**b**) Loadings plot (an enlarged version is provided in Figure S1; the abbreviations of the individual molecular descriptors are explained in Table S1). (**c**) Detailed view of the lower left region of the score plot, where mainly sensitizers are observed to form a line of data points. These sensitizers are aliphatic, monohalogenated hydrocarbons that differ primarily by chain length and halogen atom type. (**d**) Detailed view of the upper right part of the score plot, where mainly non-sensitizing compounds are located, characterized by high molecular weight, aromaticity and a large topological polar surface area.

The merged LLNA data set contains 572 skin sensitizers and 844 non-sensitizers. As shown in Figure 5a, non-sensitizers cover a broader chemical space than sensitizers. A substantial number of non-sensitizers are of higher molecular weight than sensitizers and have a stronger aromatic character and larger topological polar surface area (Figure 5a,d). A cluster of skin sensitizers and non-sensitizers with long aliphatic and halogenated chains was identified, observed as a diagonal line in the lower left of the score plot (Figure 5a,c). Interestingly, the compounds of this cluster can only be discriminated in the “MOE 2D” descriptor space but not in the Morgan2 fingerprint space, since molecules with identical halogen substitution but differing chain lengths can result in identical Morgan2 fingerprints.

### 2.3. Model Development

Prior to model development, the merged LLNA data set was divided into a training (80%) and test (20%) set (Table 3; see Methods for details). All possible combinations of machine learning approaches (random forest (RF) and support vector machine (SVM)) with up to two different sets of molecular descriptors (including molecular fingerprints) were systematically explored (Table 4). One type of descriptors to highlight is a new fingerprint that we derive from the “Protein binding alerts for skin sensitization by OASIS” profiler implemented in the OECD toolbox [29]. This profiler assigns compounds to eleven mechanistic domains associated with skin sensitization, five of which are represented by more than 20 instances in the training set (i.e., Michael addition, S_N_2 reaction, Schiff base formation, acylation, and nucleophilic addition). The new fingerprint encodes the presence or absence of alerts matching one or several of these five mechanistic domains.

For any combination of machine learning algorithm and descriptor set(s), optimum hyperparameters were identified via a grid search (Table 5). The grid search was performed within the framework of a 10-fold cross-validation, with Matthews correlation coefficient (MCC) [40] used as the scoring parameter. 

The outcomes of this grid search are summarized in Table S3. It can be seen that similar hyperparameters tend to be selected by models based on related types and sets of molecular descriptors. No strong preferences for specific hyperparameter values are apparent. This is likely related to the fact that, within a broad value space, the hyperparameters only had a minor impact on model performance.

### 2.4. Model Performance 

#### 2.4.1. Measures for the Evaluation of Model Performance

Eight different measures were applied to describe the performance of the classifiers:

- Matthews correlation coefficient (MCC), which is regarded to be one of the best measures of binary classification performance. It is robust against data imbalance and considers the proportion of all four cases of predictions (i.e., true positive, false positive, true negative and false negative predictions). Note that MCC values range from −1 to + 1. A value of + 1 indicates perfect prediction, whereas a value of −1 indicates a prediction that is in total disagreement. A value of 0 indicates a performance which is equal to random.
- ACC, which has been most commonly used by others to measure the performance of models for the prediction of the skin sensitization potential. It is defined as the proportion of correct predictions within all predictions made.
- Area under the receiver operating characteristic curve (AUC), which in this case quantifies the ability to correctly rank compounds according to their skin sensitization potential. The AUC does not rely on a decision threshold.
- Sensitivity (Se), which in this case quantifies the proportion of correctly identified skin sensitizers.
- Specificity (Sp), which in this case quantifies the proportion of correctly predicted non-sensitizers.
- Positive predictive value (PPV), which reports the proportion of true positive predictions among all positive predictions.
- Negative predictive value (NPV), which reports the proportion of true negative predictions among all negative predictions.
- CCR, which is the mean of Se and Sp.

#### 2.4.2. Model Performance During Cross-Validation

Depending on the combination of machine learning algorithm (RF or SVM) and descriptor set(s) used, MCC values ranged from 0.27 to 0.55, ACC values from 0.66 to 0.78, and AUC values from 0.63 to 0.84 (Table 4). The machine learning algorithms had only a minor impact on model performance. The average MCC values obtained by RFs and SVMs were 0.45 and 0.48, respectively. Nevertheless, the twelve predictors that obtained the highest MCC values are all based on SVMs. Most of the observed variation in performance stemmed from the use of different descriptor sets.

The best performance during cross-validation was obtained by the SVM_MOE2D+OASIS model. This model yielded an MCC, ACC and AUC of 0.55, 0.78 and 0.83, respectively. The best model based on a single set of descriptors was the SVM_PaDEL model. It reached an MCC, ACC and AUC of 0.50, 0.75 and 0.83, respectively. However, its lead over the corresponding RF model and other models based on a single set of descriptors was small. For example, the best model based on a single type of molecular fingerprint, RF_MACCS, obtained an MCC, ACC and AUC of 0.47, 0.75 and 0.81, respectively. Models based on either machine learning algorithm in combination with “MOE 2D” descriptors or MACCS fingerprints yielded comparable performance. Reduction of the full MOE2D descriptor set to the subset of 53 interpretable MOE descriptors (previously used for analyzing the chemical space coverage) led to a decline in MCC values by a maximum of 0.04. Caution needs to be exercised when interpreting these small differences in performance because of the variance observed during cross-validation. For example, for the SVM_MOE2D_53 model, the standard deviation observed for the MCC during cross-validation was 0.069.

In most cases, the combination of two sets of molecular descriptors was beneficial to model performance. Exceptions include models based on combinations of two sets of descriptors of the same type (e.g., Morgan2 and MACCS fingerprints). These did not outperform the best models based on a single set of descriptors. Also, combinations of 0D/1D/2D molecular descriptors with fingerprints did not consistently outperform models based on a single set of descriptors, albeit nine out of twelve models with MCC values greater than or equal to 0.5 are models combining non-binary molecular descriptors (i.e., MOE2D or PaDEL) with molecular fingerprints. Tables S4 and S5 provide a comprehensive overview of the impact of different combinations of descriptor sets on model performance.

Good performance was also obtained by models generated using non-commercial software only. For example, the SVM_PaDEL+OASIS model obtained MCC, ACC and AUC values of 0.50, 0.75 and 0.83, respectively. With few exceptions, the OASIS fingerprint contributed positively to the performance of models. For instance, adding the OASIS fingerprint to the SVM_MOE2D model led to an increase of the MCC, ACC and AUC by 0.07, 0.04 and 0.01, respectively. Interestingly, with a total of just 84 bits, the RF_PaDEL−Est+OASIS model reached a level of performance that is comparable with that of more complex models (MCC 0.48; ACC 0.75; AUC 0.80). However, when used on its own, the OASIS fingerprint is not sufficient for good classification performance: the RF_OASIS and SVM_OASIS models obtained the lowest MCC values across all models (i.e., 0.27 and 0.29, respectively).

#### 2.4.3. In-Depth Analysis of Selected Models within the Cross-Validation Framework

Based on the cross-validation results, five of the most interesting models were selected for additional studies:

- SVM_MOE2D+OASIS: the model with highest MCC.
- SVM_PaDEL+OASIS: a model performing comparable to the SVM_MOE2D+OASIS and based on freely available software only.
- SVM_PaDEL: the best model based on a single set of molecular descriptors.
- RF_MACCS: the best model based on a single set of molecular fingerprints.
- SVM_PaDEL+MACCS: a model with good performance, combining the descriptor sets used by the above two models.

Within the above-mentioned 10-fold cross-validation framework, we first analyzed how the coverage of the query molecules by the training data affects model performance. For this analysis we calculated the similarity between the individual query molecules and the one, three and five-nearest neighbors in the training set. Two similarity measures were explored: Tanimoto coefficients in the MACCS fingerprint space and negative Euclidean distances in the PaDEL descriptor space. The latter did not correlate well with molecular similarity (likely caused by noise related to the large number of molecular descriptors considered in this approach; Figure S2 and Table S6), for which reason we decided to go ahead with the fingerprint-based distance measure.

**Figure 6:**
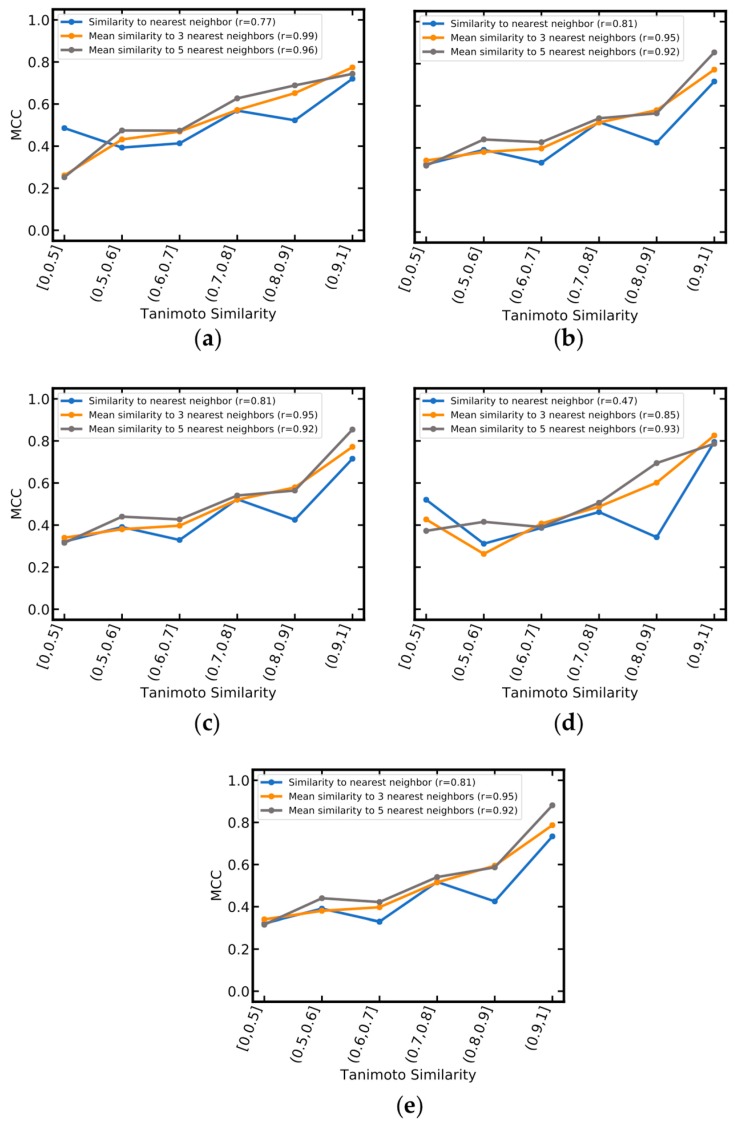
Matthews correlation coefficient (MCC) as a function of molecular similarity between the query compounds and the one, three and five nearest neighbors in the training data (calculated as averaged Tanimoto coefficients based on MACCS fingerprints). (**a**) SVM_MOE2D+OASIS; (**b**) SVM_PaDEL+OASIS; (**c**) SVM_PaDEL; (**d**) RF_MACCS; (**e**) SVM_PaDEL+MACCS. Pearson correlation coefficients are reported in brackets in the figure legends. The number of compounds in each bin is summarized in Table S7.

For all five models, a direct linear relationship was observed between MCC values and molecular similarity. The relationship was consistent when considering different numbers of nearest neighbors in the training data but tended to be more robust when taking more (i.e., 5) nearest neighbors into account (Pearson correlation coefficient between 0.92 and 0.96 when considering five nearest neighbors). As shown in Figure 6, for compounds dissimilar to those present in the training data (defined by Tanimoto coefficients averaged over the five nearest neighbors of 0.5 or lower), MCC values were below or around 0.4 for all five models. For compounds structurally related to the training data (defined by Tanimoto coefficients of 0.7 or higher), MCC values were at least 0.5 or higher.

**Figure 7:**
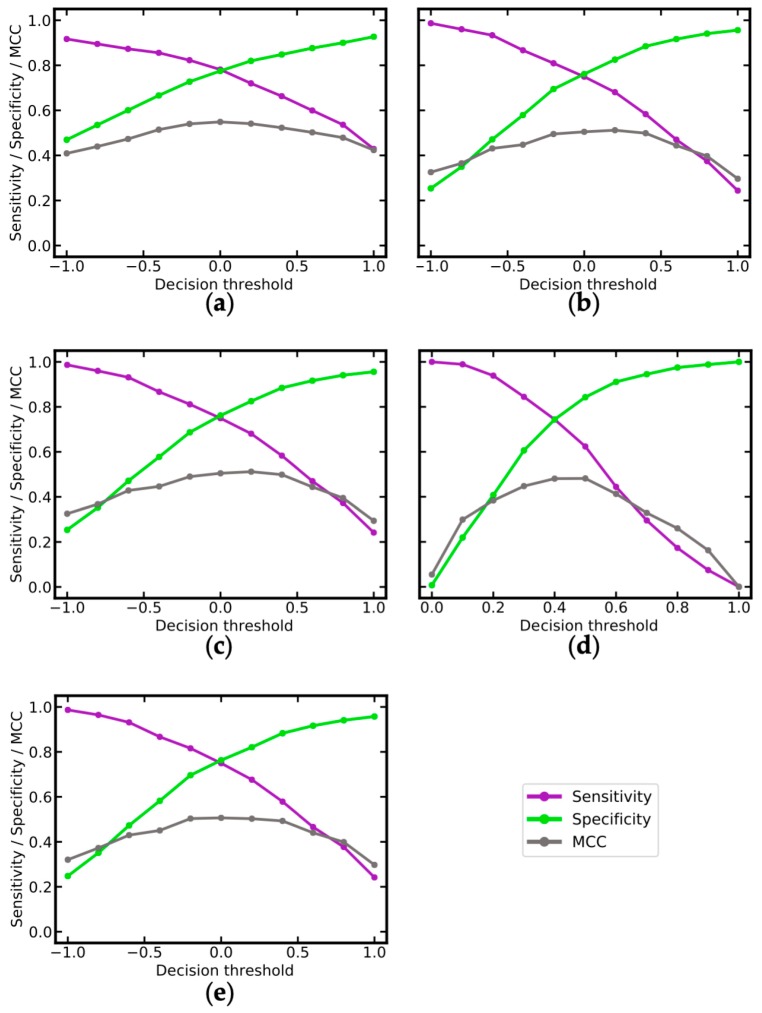
Matthews correlation coefficient (MCC), sensitivity and specificity as a function of the decision threshold, for (**a**) SVM_MOE2D+OASIS; (**b**) SVM_PaDEL+OASIS; (**c**) SVM_PaDEL; (**d**) RF_MACCS; (**e**) SVM_PaDEL+MACCS. Note that different *X*-axis scales are applied to the graphs illustrating the performance of random forest (RF) and support vector machine (SVM) models.

Secondly, we investigated how changes to the decision threshold of the SVM and RF classifiers (i.e., the value above which a compound is predicted to be a sensitizer) affect the sensitivity and specificity of the models. As shown in Figure 7, both these metrics strongly depend on the selected decision threshold. This allows users to define context-dependent thresholds. For example, in scenarios where for a compound of interest any skin sensitization potential should be ruled out, users may opt for lower decision thresholds to identify any hazard. In the case of the RF_MACCS model, lowering the decision threshold to 0.3 results in a sensitivity of 0.84 and a specificity of 0.61 (Figure 7d).

**Figure 8:**
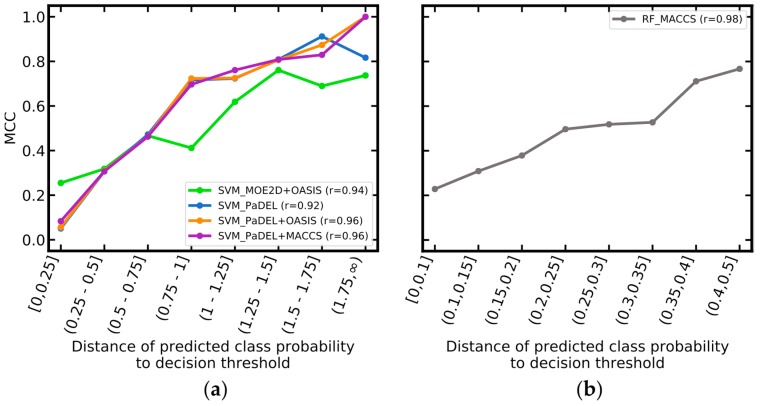
Matthews correlation coefficient (MCC) as a function of the distance between the predicted class probabilities and the decision thresholds, for the (**a**) support vector machine (SVM) models and (**b**) random forest (RF) model. The number of compounds in each bin is summarized in Table S8.

Observing the predicted class probability can be of use for assessing the reliability of a prediction: as shown in Figure 8, the reliability of predictions increases with the absolute distance between the class probability and the decision threshold. For SVM models, predictions with class probabilities more than 0.5 away from the decision threshold had averaged MCC values between 0.63 and 0.67, whereas predictions with class probabilities less than 0.5 away had averaged MCC values of just 0.20 to 0.29. For the RF_MACCS model, predictions with class probabilities more than 0.35 away from the decision threshold had MCC values above 0.6, whereas predictions with class probabilities closer than 0.15 to the decision threshold had MCCs below 0.4. For the five investigated models, the Pearson correlation coefficients for this relationship were between 0.92 and 0.98.

**Figure 9:**
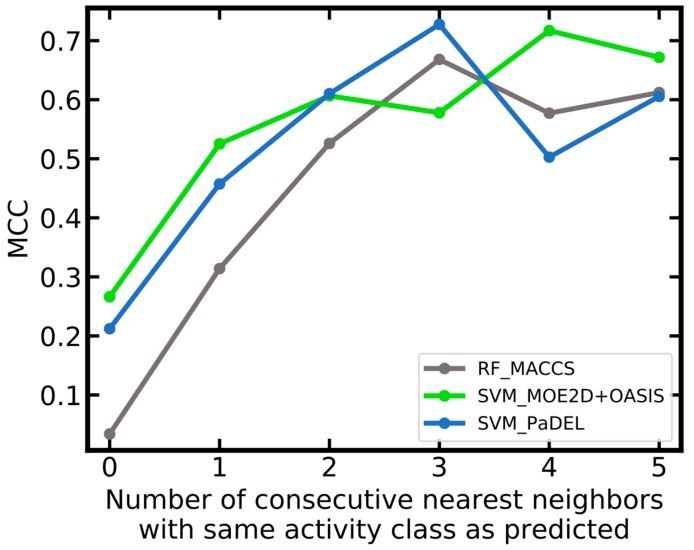
Matthews correlation coefficient (MCC) as a function of the number of consecutive nearest neighbors in the training data that are of the same activity class as the predicted class for a compound of interest (molecular similarity quantified as Tanimoto coefficient based on MACCS fingerprints). The number of compounds in each bin is summarized in Table S9. The graphs for SVM_PaDEL+OASIS and SVM_PaDEL+MACCS are not shown because they are (almost) identical with that of SVM_PaDEL and would overlap.

As a further way of analyzing the data, we looked into the reliability of predictions as a function of the number of consecutive nearest neighbors in the training data that are of the same activity class as the one predicted for a compound of interest. From Figure 9, it can be seen that predictions are particularly reliable if the three nearest neighbors in the training data are of the identical class as the class predicted for a compound of interest. The strongest correlation is observed for the RF_MACCS model. For this model the MCC is close to zero for compounds where the predicted class is in conflict with the class assigned to the nearest neighbor. In contrast, the MCC is above 0.6 for compounds where the predicted class and the classes assigned to the three nearest neighbors are identical.

#### 2.4.4. Performance of Selected Models on the Test Set

The performance of the five selected models was tested on holdout data. All models were stable, with only minor losses in MCC, ACC and AUC when compared to the results from cross-validation (Table 6). The largest losses in performance were observed for the RF_MACCS model, with MCC and ACC values decreased by 0.06 and 0.03, respectively (AUC however +0.01).

By defining the applicability domain of the models to include any compounds with a minimum Tanimoto coefficient of 0.75 averaged over the five-nearest neighbors in the training set (based on MACCS fingerprints), MCC values increased, in the case of the RF_MACCS model from 0.41 to 0.59. However, at the same time the coverage of the test set is reduced, in the case of RF_MACCS to 28%.

Defining the applicability domain with a cutoff of 0.50 rather than 0.75 led to only minor performance improvements compared to the model without applicability domain definition. This is related to the fact that only approximately 3% of the compounds of the test set are that dissimilar to the compounds in the training data. However, predictions for these compounds are unreliable (MCC values 0.2 or lower). Therefore, it is important to observe the applicability domain of the individual models.

Besides the applicability domain definition, users are advised to consider two additional types of information when judging the reliability of a prediction: (i) the distance between the predicted class probability from the decision threshold and (ii) the number of consecutive nearest neighbors that are of the same activity class than the class predicted for a compound of interest.

Larger distances of the class probability to the decision threshold indicate higher reliability of the prediction. For example, when considering only predictions with class probabilities 0.35 or further away from the decision threshold, the MCC of the RF_MACCS model increases from 0.41 to 0.78 (this covers 23% of the test set; Table 7). Likewise, for the SVM models, MCC values increase from approximately 0.5 to a maximum of 0.78 when considering predictions only if their class probability is 1.25 or further away from the decision threshold (this covers 12% to 37% of the compounds in the test set).

Predictions for query molecules that are consistent with the class assigned to the *k*-nearest neighbors in the training data are more reliable than for those that are in conflict. This is also confirmed by the results obtained for the test set (Table 8): Predictions that are in disagreement with the activity class of the nearest neighbor resulted in MCC and ACC values no higher than 0.13 and 0.56, respectively. MCC and ACC values increase to a maximum of 0.98 and 0.99 when considering predictions only if they are consistent with three or more nearest neighbors.

#### 2.4.5. Comparison of Model Performance to that of Existing Models

Major caveats must be considered when attempting to directly compare the performance reported for existing models with those presented in this work. Not only do the underlying training and test sets differ substantially, but also the protocols used for performance evaluation and the definitions of the models’ applicability domains. Roughly summarized, Alves et al. reported their predictor of binary LLNA outcomes to yield a CCR of 0.77 during external cross-validation [28]. Di et al. reported their best global model for the binary prediction of LLNA outcomes, a SVM model based on PaDEL-Ext descriptors (Ext-SVM), to have yielded an ACC of 0.84 during cross-validation and an ACC of 0.81 on their test set (when considering the applicability domain according to their definition) [22]. In comparison, our best model (SVM_MOE2D+OASIS) yielded a CCR of 0.78 and identical ACC during cross-validation (MCC 0.55), without consideration of the applicability domain. On the test set, the SVM_MOE2D+OASIS model obtained a CCR of 0.76 and an MCC of 0.52. In this case, the consideration of the applicability domain of the model (defined as including any compound with a mean Tanimoto similarity to the five nearest neighbors in the training set of 0.50 or higher) did not yield a further improvement of performance. The SVM_PaDEL and RF_MACCS models, which are available via a public web service, yielded comparable CCR values (0.74 and 0.70 without consideration of the applicability domain; 0.75 and 0.71 with consideration of the applicability domain, respectively). The latter model has the additional benefit of being based on a fingerprint with a length of only 166 bits.

### 2.5. Skin Doctor Web Service

The final RF_MACCS and SVM_PaDEL models, trained not on the cross-validation data set but on the complete, preprocessed data set (1416 and 1388 compounds, depending on the number of compounds for which descriptors could be successfully calculated) are provided via the New E-Resource for Drug Discovery (NERDD) [41]. Queries can either be directly drawn or uploaded in different formats. Users may change the default decision threshold to steer the model’s sensitivity and specificity. Results are presented in a tabular overview and can be exported as a CSV file. For each query they include information on (i) whether or not the query is within the applicability domain of the model, (ii) the predicted activity classes, (iii) distances from the selected decision threshold, (iv) mean similarity between the query compound and the five-nearest neighbors of the training set and (v) number of consecutive nearest neighbors in the training data of which the activity label is consistent with that of the prediction. The analysis and visualization of the corresponding effects presented in this work may be used as guidance to choose the required confidence in the prediction, being aware of the corresponding effects on the model’s applicability domain and the requirements for similarity.

Predictions are flagged with reliability warnings (a) if the mean similarity between the compound of interest and the five nearest neighbors is less than 0.5, or (b) if the predictions are in conflict with the activity of the nearest neighbor in the training data, or (c) if the distance to the decision threshold is small (0.15 for the RF_MACCS model; 0.5 for the SVM_PaDEL model).

## 3. Materials and Methods

### 3.1. Data Preparation

The LLNA data set compiled by Alves et al. was downloaded from Chembench. Binary class labels (i.e., “sensitizer”, “non-sensitizer”) were obtained from the binary property “LLNA result” and not altered. The LLNA data set of Di et al. was obtained from the supporting information associated with their publication [22]. Binary class labels (i.e., “sensitizer”, “non-sensitizer”) were assigned based on the information provided by the property “class”: any compounds with the value “negative” were assigned the label “non-sensitizer”; any compounds with the value “weak”, “moderate”, “strong” or “extreme” were assigned the label “sensitizer”. Reference data sets of cosmetic substances and ingredients (hereafter “cosmetics”), approved drugs and pesticides were obtained from the EU CosIng database, Drugbank and EU pesticides database.

All data sets were processed individually according to the following protocol: Any counterions were removed and the remaining molecular structures neutralized as described in the work of Stork et al. [42]. Tautomers were standardized with the “TautomerCanonicalizer” method implemented in the “tautomer” class of MolVS [43]. This was followed by a deduplication of molecules based on canonicalized SMILES. Stereochemical information was disregarded at this point, leading to conflicting activity labels for one compound (which had different activity labels assigned to the two enantiomers). This compound was removed from the data set.

A merged LLNA data set based on the LLNA data sets of Alves et al. and Di et al. was generated by filtering duplicates based on canonical SMILES and removing any compounds with contradicting class labels.

### 3.2. Descriptor Calculation

Molecular descriptors were computed with the Molecular Operating Environment (MOE) [36] (“MOE descriptors”), RDKit [39] (Morgan and MACCS fingerprints) and PaDEL [37,38] (“PaDEL descriptors” as well as the molecular fingerprints “PaDEL-Est” and “PaDEL-Ext”). “MOE 2D” descriptors were calculated with default settings. Morgan fingerprints (2048 bits) were calculated with a radius of 2. MACCS fingerprints were calculated with default settings. Also, the PaDEL descriptors were calculated with default settings, with the exception of a maximum allowed runtime of 1000 s per molecule. Structural alerts were computed with the OECD toolbox [29] using the “Protein binding alerts for skin sensitization by OASIS” profiler with default settings. All non-binary descriptors were scaled to unit variance and their mean shifted to zero prior to model building and data analysis using the StandardScaler of scikit-learn [44].

### 3.3. Data Analysis

PCA was conducted with scikit-learn based on a subset of 53 physically meaningful, scaled “MOE 2D” descriptors (Table S1). RDKit was employed for generating Murcko scaffolds and calculating molecular similarity.

### 3.4. Compilation of Data Sets for Model Development

The merged LLNA data set was divided into a training set (80%) and a test set (20%) by stratified splitting with the train_test_split function of the model_selection module of scikit-learn (data shuffling prior to data set splitting enabled). This procedure was assigned a random state of 43.

### 3.5. Model Generation

Models were generated with scikit-learn and a random_state value of 43. Default settings were applied, with the exception of class_weight set to “balanced” for both RF and SVM. SVMs were used with a radial basis function (RBF) kernel. Optimal settings for n_estimators and max_features (RF models) and C and gamma (SVM models) were derived during grid search.

### 3.6. Hardware and Software

All calculations were performed on Linux workstations running openSUSE Leap 15.0 and equipped with Intel i5 processors (3.2 GHz) and 16 GB of main memory.

## 4. Conclusions

Building on the works of Alves et al. and Di et al., we have compiled a collection of 1416 compounds annotated with binary LLNA outcomes. To our knowledge, this is the largest LLNA data set that has been used for the development of models predicting the skin sensitization potential of small organic molecules. As we show by chemical space analysis, those areas most densely populated by cosmetics, approved drugs and pesticides are also well covered by this new LLNA data set. The fraction of compounds covered by structurally related compounds in the new LLNA data set is much higher for cosmetics (30%) than for approved drugs (10%) and pesticides (13%). Therefore, the models are applicable to many compounds typically used in cosmetic products. However, there are chemical classes of drugs and cosmetics that are not adequately represented by the available LLNA data. This emphasizes the importance of considering the applicability domain of models.

An interesting observation to make was that a cluster of skin sensitizers and non-sensitizers with long aliphatic and halogenated chains could only be discriminated in the “MOE 2D” descriptor space but not in the Morgan2 fingerprint space, which should be taken into consideration for model building. The best models derived from the new LLNA data set obtained MCC and ACC values of up to 0.55 and 0.78 during cross-validation and of up to 0.52 and 0.76 on holdout data, respectively. Importantly, some of the models based entirely on free software and/or molecular descriptors of low complexity yielded comparable performance. We identified the RF_MACCS and SVM_PaDEL models as our favorite models, yielding MCC values of 0.41 and 0.47 on the holdout data. Comparison to existing models indicates that our models reach competitive performance. They are trained on a data set consisting of almost 3.5 times as many compounds as the one used by Di et al. The full data set used for modeling and testing is also 42% larger than that of Alves et al. given the fact that the data set compiled by Di et al. holds in particular a diverse set of non-sensitizers not covered by Alves et al. we expect that our models, as they are based on the amalgamated data set, are more widely applicable and more reliable.

A major aspect of this work is the definition of an applicability domain for the individual models and the elaboration of means to estimate the reliability of predictions. The applicability domain was defined based on the mean similarity of a compound of interest to the five-nearest neighbors in the training data (quantified in MACCS fingerprint space). The difference between the predicted class probability and the decision threshold, as well as the number of consecutive nearest neighbors in the training data having the same activity class assigned as the one predicted for the compound of interest proved to be useful indicators of the reliability of predictions. We recommend considering predictions as reliable if all of the following conditions are met:

1. The compound of interest is within the applicability domain of the model.
2. The distance between the predicted class probability and the decision threshold is at least 0.15 for RF models and 0.5 for SVM models.
3. The predicted activity class for a compound of interest is in agreement with the class assigned to the nearest neighbor in the training data.

The public web service, available at https://nerdd.zbh.uni-hamburg.de/, provides access to the final RF_MACCS and SVM_PaDEL models (i.e., models trained on the complete LLNA data set). Users are provided detailed information on whether or not a compound of interest fulfills the three criteria itemized above. A warning is issued in case predictions are determined to be unreliable. Users may also adjust the decision threshold, allowing them, e.g., to increase the model’s sensitivity in scenarios where it is desirable to flag even substances with a low likelihood of being skin sensitizers.

We hope that the models will be well received by the scientific community and will make a contribution to the development and application of non-animal methods for the prediction of the skin sensitization potential of small organic molecules.

## Figures and Tables

**Table 1:** Overview of all data sets used in this work.

	LLNA Data Set Compiled by Alves et al.	LLNA Data Set Compiled by Di et al.	Merged LLNA Data Set	Cosmetic Substances and Ingredients Data Set	Approved Drugs Data Set	Pesticides Data Set
Data source	Chembench [30]^1^	Supporting information of Di et al. [22]	LLNA data sets of Alves et al. and Di et al.	CosIng Database [31]	“Approved Drugs” subset of DrugBank [32,33]^2^	EU Pesticides Database [34]
Number of compounds prior to data preprocessing	1000	1007	1993	5937	2352	1383
Number of compounds after data preprocessing	1000	993^3^	1416^4^ (1132/284)^5^	4643^6^	2155^7^	812^8^
Number of sensitizers	481	364	572 (457/115)^5^	*n*/a	*n*/a	*n*/a
Number of non-sensitizers	519	629	844 (675/169)^5^	*n*/a	*n*/a	*n*/a
Number of Murcko scaffolds	312	354	453	856	1158	329
Proportion of compounds without a Murcko scaffold	0.32	0.29	0.31	0.42	0.13	0.24
Proportion of singleton scaffolds	0.77	0.79	0.78	0.72	0.82	0.81

^1^ Chapel Hill, NC, United States. ^2^ Edmonton, Alberta, Canada. ^3^ Thirteen compounds were removed as part of the deduplication procedure; one compound was removed because of conflicting activity assignments. ^4^ Five hundred and sixty-seven compounds were removed as part of the deduplication procedure; ten compounds were removed because of conflicting activity assignments. ^5^ Number of compounds in the training set/test set prior to descriptor calculation. ^6^ One hundred and four compounds were removed by the salt filter because the main component could not be unambiguously identified; 26 compounds were removed due to invalid input structure; 1164 compounds were removed as part of the deduplication procedure. ^7^ Thirty-one compounds were removed by the salt filter because the main component could not be unambiguously identified; 166 compounds were removed as part of the deduplication procedure. ^8^ The SMILES notation of 893 compounds present in the EU Pesticides Database were automatically retrieved with the Chemical Identifier Resolver [35]. Six compounds were removed by the salt filter because the main component could not be identified; 13 compounds were removed due to invalid input structure; 62 compounds were removed as part of the deduplication procedure. Abbreviations: LLNA, local lymph node assay.

**Table 2:** Overlaps between the compounds contained in the LLNA data sets and the cosmetics, approved drugs and pesticides data sets.

	Number of Compounds	Data Set Compiled by Alves et al.	Data Set Compiled by Di et al.	Merged LLNA Data Set
Cosmetics	4643	324	252	387
Approved Drugs	2155	88	68	97
Pesticides	812	43	34	44

Abbreviations: LLNA, local lymph node assay.

**Table 3:** Overview of descriptor sets evaluated in this work.

Descriptor Set	Short Name	Number of Descriptors/Length of the Fingerprint	Calculated with	Number of Successfully Processed Molecules^1^	
				Training set	Test set
0D, 1D and 2D descriptors	MOE2D	206	MOE [36]; this set corresponds to all descriptors listed as “2D descriptors” in MOE	1132	284
Selection of 0D, 1D and 2D descriptors	MOE2D_53	53^2^	MOE [36]	1132	284
0D, 1D and 2D descriptors	PaDEL	1444	PaDEL [37,38]; this is the complete set of 0D, 1D and 2D descriptors implemented in PaDEL	1109	279
MACCS keys	MACCS	166	RDKit [39]	1132	284
Morgan2 fingerprints	Morgan2	2048	RDKit [39]	1132	284
OASIS skin sensitization protein binding fingerprint	OASIS	5 bit fingerprint	OECD Toolbox [29]	1128	283
PaDEL estate fingerprint	PaDEL_Est	79	PaDEL [37,38]	1132	284
PaDEL extended fingerprint	PaDEL_Ext	1024	PaDEL [37,38]	1132	284

^1^ Descriptor calculation failed for individual compounds depending on the software used. For this reason, there are marginal differences in the composition of the individual data sets used for model development. ^2^ Fifty-three manually selected, physically meaningful descriptors. A list of the selected descriptors can be found in Table S1. Abbreviations: MOE, Molecular Operating Environment.

**Table 4:** Overview of models and their performance during cross-validation.

Name	Number of Descriptors	Number of Compounds in Training Data	ACC	ACC STDEV	MCC	MCCSTDEV	AUC	CCR	Se	SP	PPV	NPV
SVM_MOE2D+OASIS	211	1128	0.78	0.054	0.55	0.109	0.83	0.78	0.77	0.78	0.71	0.83
SVM_PaDEL+MACCS	1610	1108	0.76	0.035	0.51	0.069	0.83	0.76	0.75	0.76	0.69	0.82
SVM_PaDEL+Morgan2	3492	1108	0.76	0.036	0.51	0.078	0.82	0.75	0.66	0.83	0.73	0.78
SVM_PaDEL+PaDEL-Ext	2468	1109	0.76	0.039	0.51	0.075	0.84	0.76	0.74	0.78	0.7	0.81
SVM_MOE2D+MACCS	372	1132	0.76	0.047	0.5	0.096	0.81	0.74	0.68	0.81	0.71	0.79
SVM_MOE2D+Morgan2	2254	1132	0.75	0.041	0.5	0.081	0.83	0.75	0.77	0.73	0.66	0.83
SVM_MOE2D+PaDEL	1680	1109	0.76	0.039	0.5	0.079	0.83	0.75	0.74	0.77	0.69	0.81
SVM_MOE2D+PaDEL-Est	285	1132	0.76	0.039	0.5	0.081	0.81	0.75	0.68	0.81	0.71	0.79
SVM_MOE2D+PaDEL-Ext	1230	1132	0.75	0.054	0.5	0.105	0.83	0.75	0.75	0.76	0.68	0.81
SVM_PaDEL	1444	1109	0.75	0.038	0.5	0.075	0.83	0.75	0.75	0.75	0.68	0.81
SVM_PaDEL+OASIS	1449	1109	0.75	0.038	0.5	0.075	0.83	0.75	0.75	0.75	0.68	0.81
SVM_PaDEL+PaDEL-Est	1523	1109	0.75	0.038	0.5	0.075	0.83	0.75	0.75	0.75	0.68	0.81
RF_PaDEL+MACCS	1610	1108	0.76	0.018	0.49	0.037	0.82	0.73	0.62	0.85	0.74	0.77
RF_PaDEL+Morgan2	3492	1108	0.76	0.02	0.49	0.042	0.82	0.74	0.64	0.84	0.73	0.77
RF_PaDEL+OASIS	1449	1109	0.76	0.02	0.49	0.043	0.82	0.74	0.62	0.85	0.74	0.77
RF_PaDEL+PaDEL-Ext	2468	1109	0.76	0.022	0.49	0.048	0.82	0.73	0.61	0.86	0.75	0.76
SVM_PaDEL-Est+MACCS	245	1132	0.75	0.051	0.49	0.106	0.81	0.74	0.69	0.8	0.7	0.79
RF_MOE2D+PaDEL	1680	1109	0.75	0.034	0.48	0.072	0.83	0.73	0.62	0.84	0.73	0.77
RF_Morgan2+PaDEL-Est	2127	1132	0.76	0.033	0.48	0.071	0.82	0.73	0.63	0.84	0.73	0.77
RF_PaDEL	1444	1109	0.75	0.015	0.48	0.033	0.82	0.73	0.62	0.84	0.73	0.76
RF_PaDEL-Est+OASIS	84	1128	0.75	0.043	0.48	0.091	0.8	0.74	0.65	0.82	0.72	0.78
SVM_MACCS+OASIS	171	1128	0.75	0.047	0.48	0.102	0.82	0.74	0.69	0.79	0.69	0.79
SVM_MOE2D	206	1132	0.74	0.037	0.48	0.067	0.82	0.74	0.75	0.74	0.66	0.82
SVM_Morgan2+PaDEL-Ext	3072	1132	0.75	0.044	0.48	0.09	0.82	0.74	0.68	0.8	0.7	0.79
RF_MACCS	166	1132	0.75	0.039	0.47	0.088	0.81	0.73	0.61	0.84	0.73	0.76
RF_MACCS+OASIS	171	1128	0.75	0.034	0.47	0.074	0.8	0.73	0.6	0.85	0.74	0.76
RF_PaDEL+PaDEL-Est	1523	1109	0.75	0.028	0.47	0.06	0.83	0.73	0.61	0.85	0.73	0.76
SVM_MACCS	166	1132	0.74	0.057	0.47	0.12	0.81	0.73	0.69	0.78	0.68	0.79
SVM_PaDEL-Est+OASIS	84	1128	0.74	0.048	0.47	0.099	0.8	0.74	0.71	0.76	0.67	0.8
SVM_PaDEL-Est+PaDEL-Ext	1103	1132	0.74	0.039	0.47	0.08	0.81	0.74	0.7	0.78	0.68	0.79
SVM_PaDEL-Ext	1024	1132	0.74	0.046	0.47	0.093	0.81	0.73	0.7	0.77	0.68	0.79
SVM_PaDEL-Ext+OASIS	1029	1128	0.74	0.036	0.47	0.072	0.82	0.74	0.7	0.77	0.68	0.79
RF_MOE2D+Morgan2	2254	1132	0.74	0.033	0.46	0.071	0.81	0.72	0.62	0.82	0.71	0.76
RF_PaDEL-Est+MACCS	245	1132	0.75	0.045	0.46	0.1	0.81	0.72	0.59	0.85	0.73	0.76
RF_Morgan2	2048	1132	0.74	0.039	0.46	0.081	0.81	0.73	0.64	0.81	0.7	0.77
SVM_Morgan2+MACCS	2214	1132	0.74	0.058	0.46	0.117	0.8	0.73	0.68	0.78	0.68	0.78
SVM_PaDEL-Ext+MACCS	1190	1132	0.74	0.047	0.46	0.097	0.81	0.73	0.68	0.77	0.68	0.78
RF_MOE2D+OASIS	211	1128	0.74	0.041	0.45	0.09	0.81	0.71	0.6	0.83	0.71	0.75
RF_MOE2D+PaDEL-Est	285	1132	0.74	0.032	0.45	0.07	0.81	0.72	0.6	0.84	0.72	0.75
RF_MOE2D+PaDEL-Ext	1230	1132	0.74	0.017	0.45	0.037	0.82	0.72	0.58	0.85	0.73	0.75
RF_MOE2D	206	1132	0.73	0.036	0.44	0.078	0.81	0.71	0.59	0.83	0.71	0.75
RF_MOE2D+MACCS	372	1132	0.73	0.033	0.44	0.072	0.81	0.71	0.58	0.84	0.71	0.75
RF_Morgan2+MACCS	2214	1132	0.73	0.039	0.44	0.086	0.8	0.72	0.63	0.8	0.68	0.76
RF_Morgan2+OASIS	2053	1128	0.74	0.029	0.44	0.063	0.82	0.71	0.59	0.83	0.71	0.75
RF_Morgan2+PaDEL-Ext	3072	1132	0.73	0.036	0.44	0.081	0.81	0.71	0.56	0.85	0.72	0.74
SVM_MOE2D53	53	1132	0.71	0.037	0.44	0.069	0.78	0.72	0.76	0.68	0.62	0.81
SVM_PaDEL-Est	79	1132	0.72	0.037	0.44	0.073	0.77	0.72	0.71	0.73	0.64	0.79
RF_PaDEL-Est	79	1132	0.73	0.022	0.43	0.042	0.77	0.71	0.64	0.79	0.67	0.76
RF_PaDEL-Ext+MACCS	1190	1132	0.73	0.037	0.43	0.081	0.81	0.7	0.55	0.85	0.72	0.74
RF_PaDEL-Ext+OASIS	1029	1128	0.73	0.033	0.43	0.072	0.8	0.7	0.57	0.84	0.71	0.74
RF_PaDEL-Ext+PaDEL-Est	1103	1132	0.73	0.034	0.43	0.074	0.8	0.7	0.56	0.85	0.72	0.74
SVM_Morgan2+OASIS	2053	1128	0.73	0.038	0.43	0.089	0.8	0.69	0.51	0.88	0.75	0.73
SVM_Morgan2+PaDEL-Est	2127	1132	0.72	0.035	0.43	0.064	0.79	0.72	0.69	0.75	0.65	0.78
RF_MOE2D53	53	1132	0.73	0.039	0.42	0.086	0.78	0.7	0.58	0.83	0.69	0.74
RF_PaDEL-Ext	1024	1132	0.72	0.039	0.42	0.088	0.79	0.7	0.55	0.84	0.71	0.73
SVM_Morgan2	2048	1132	0.72	0.031	0.39	0.072	0.8	0.68	0.49	0.87	0.72	0.71
SVM_OASIS	5	1128	0.67	0.064	0.29	0.151	0.63	0.62	0.37	0.87	0.68	0.67
RF_OASIS	5	1128	0.66	0.054	0.27	0.122	0.64	0.63	0.43	0.82	0.62	0.68

Abbreviations: ACC, accuracy; AUC, area under the receiver operating characteristic curve; CCR, correct classification rate; MCC, Matthews correlation coefficient; NPV, negative predictive value; PPV, positive predictive value; Se, sensitivity; Sp, specificity; STDEV, standard deviation.

**Table 5:** Overview of hyperparameters optimized by grid search.

Machine Learning Approach	Parameter	Explored Values
RF	*n*_estimators ^1^	10, 50, 100, 250, 500, 1000
	max_features ^2^	‘sqrt’, 0.2, 0.4, 0.6, 0.8, None
SVM	C ^3^	0.01, 0.1, 1, 10, 100, 1000
	gamma ^4^	1, 0.1, 0.01, 0.001, 0.0001, 0.00001

^1^ Number of prediction trees. ^2^ Maximum depth of each tree. ^3^ Penalty parameter C of the error term. ^4^ Coefficient for the radial basis function (rbf) kernel. Abbreviations: RF, random forest; SVM, support vector machine.

**Table 6:** Performance of selected models on the test set.

NAME	Mean Tanimoto Similarity to the Five Nearest Neighbors	Number of Compounds	ACC	MCC	AUC	CCR	Se	Sp	PPV	NPV
RF_MACCS	≥0	284	0.72	0.41	0.82	0.70	0.57	0.82	0.69	0.74
RF_MACCS	≥0.5	273	0.73	0.43	0.82	0.71	0.6	0.82	0.69	0.75
RF_MACCS	≥0.75	79	0.78	0.59	0.91	0.81	0.89	0.73	0.64	0.92
RF_MACCS	<0.5	11	0.45	−0.29	0.60	0.42	0.00	0.83	0.00	0.50
SVM_MOE_2D+OASIS	≥0	283	0.76	0.52	0.83	0.76	0.81	0.72	0.66	0.85
SVM_MOE_2D+OASIS	≥0.5	273	0.76	0.53	0.84	0.77	0.82	0.72	0.67	0.86
SVM_MOE_2D+OASIS	≥0.75	79	0.81	0.64	0.89	0.84	0.93	0.75	0.67	0.95
SVM_MOE2D+OASIS	<0.5	10	0.60	0.20	0.60	0.60	0.60	0.60	0.60	0.60
SVM_PaDEL	≥0	279	0.74	0.47	0.82	0.74	0.76	0.72	0.65	0.82
SVM_PaDEL	≥0.5	269	0.74	0.49	0.83	0.75	0.77	0.73	0.65	0.83
SVM_PaDEL	≥0.75	79	0.80	0.63	0.89	0.83	0.93	0.73	0.65	0.95
SVM_PaDEL	<0.5	10	0.60	0.20	0.56	0.60	0.60	0.60	0.60	0.60
SVM_PaDEL+MACCS	≥0	279	0.75	0.50	0.82	0.75	0.78	0.73	0.66	0.83
SVM_PaDEL+MACCS	≥0.5	269	0.75	0.51	0.83	0.76	0.79	0.73	0.66	0.84
SVM_PaDEL+MACCS	≥0.75	79	0.80	0.63	0.89	0.83	0.93	0.73	0.65	0.95
SVM_PaDEL+MACCS	<0.5	10	0.60	0.20	0.56	0.60	0.60	0.60	0.60	0.60
SVM_PaDEL+OASIS	≥0	279	0.74	0.48	0.82	0.74	0.76	0.73	0.65	0.82
SVM_PaDEL+OASIS	≥0.5	271	0.75	0.49	0.83	0.75	0.77	0.73	0.65	0.83
SVM_PaDEL+OASIS	≥0.75	79	0.80	0.63	0.89	0.83	0.93	0.73	0.65	0.95
SVM_PaDEL+OASIS	<0.5	10	0.60	0.20	0.56	0.60	0.60	0.6	0.60	0.60

Abbreviations: ACC, accuracy; AUC, area under the receiver operating characteristic curve; CCR, correct classification rate; MCC, Matthews correlation coefficient; NPV, negative predictive value; PPV, positive predictive value; Se, sensitivity; Sp, specificity.

**Table 7:** Test set performance as a function of the distance of predicted class probabilities from the decision threshold.

Name	Distance to Decision Threshold ^1^	Number of Compounds	ACC	MCC	AUC	CCR	Se	Sp	PPV	NPV
RF-MACCS	≥0.15	175	0.85	0.67	0.46	0.84	0.81	0.87	0.76	0.90
RF-MACCS	≥0.35	66	0.91	0.78	0.42	0.89	0.85	0.93	0.85	0.93
RF-MACCS	<0.15	109	0.51	0.04	0.42	0.52	0.32	0.72	0.55	0.50
SVM_MOE2D+OASIS	≥0.5	203	0.82	0.64	0.42	0.83	0.88	0.78	0.73	0.90
SVM_MOE2D+OASIS	≥1.25	106	0.89	0.76	0.41	0.89	0.89	0.88	0.81	0.94
SVM_MOE2D+OASIS	<0.50	80	0.60	0.20	0.52	0.60	0.62	0.58	0.50	0.70
SVM_PaDEL	≥0.5	183	0.80	0.61	0.48	0.81	0.86	0.76	0.71	0.89
SVM_PaDEL	≥1.25	34	0.88	0.78	0.45	0.91	1.00	0.82	0.75	1.00
SVM_PaDEL	<0.50	96	0.61	0.21	0.36	0.60	0.55	0.66	0.51	0.69
SVM_PaDEL+MACCS	≥0.5	183	0.80	0.62	0.49	0.82	0.88	0.75	0.71	0.90
SVM_PaDEL+MACCS	≥1.25	37	0.86	0.75	0.52	0.9	1.00	0.80	0.71	1.00
SVM_PaDEL+MACCS	<0.50	96	0.65	0.27	0.39	0.63	0.58	0.69	0.55	0.71
SVM_PaDEL+OASIS	≥0.5	183	0.80	0.61	0.49	0.81	0.86	0.76	0.71	0.89
SVM_PaDEL+OASIS	≥1.25	34	0.88	0.78	0.45	0.91	1.00	0.82	0.75	1.00
SVM_PaDEL+OASIS	<0.50	96	0.62	0.22	0.37	0.61	0.55	0.67	0.52	0.70

^1^ Distance of predicted class probabilities from the decision threshold. Abbreviations: ACC, accuracy; AUC, area under the receiver operating characteristic curve; CCR, correct classification rate; MCC, Matthews correlation coefficient; NPV, negative predictive value; PPV, positive predictive value; Se, sensitivity; Sp, specificity.

**Table 8:** Test set performance as a function of the number of consecutive nearest neighbors with class assignments consistent with the predicted class.

Name	Number of Concordant Neighbors^1^	Number of Compounds	ACC	MCC	AUC	CCR	Se	Sp	PPV	NPV
RF_MACCS	0	87	0.33	-0.35	0.32	0.33	0.19	0.48	0.26	0.38
RF_MACCS	≥1	197	0.89	0.77	0.97	0.87	0.81	0.94	0.89	0.89
RF_MACCS	≥2	147	0.96	0.90	1.00	0.94	0.89	0.99	0.98	0.95
RF_MACCS	≥3	113	0.99	0.98	1.00	0.98	0.97	1.00	1.00	0.99
SVM_MOE2D+OASIS	0	85	0.56	0.13	0.56	0.57	0.62	0.51	0.55	0.58
SVM_MOE2D+OASIS	≥1	198	0.84	0.69	0.94	0.85	0.92	0.79	0.72	0.94
SVM_MOE2D+OASIS	≥2	146	0.91	0.81	0.99	0.92	0.95	0.89	0.79	0.98
SVM_MOE2D+OASIS	≥3	115	0.91	0.80	0.99	0.92	0.94	0.90	0.79	0.97
SVM_PaDEL	0	86	0.53	0.07	0.52	0.54	0.56	0.51	0.51	0.56
SVM_PaDEL	≥1	193	0.83	0.66	0.92	0.84	0.87	0.8	0.72	0.92
SVM_PaDEL	≥2	147	0.89	0.78	0.96	0.91	0.96	0.86	0.76	0.98
SVM_PaDEL	≥3	113	0.90	0.79	0.97	0.92	0.97	0.88	0.76	0.99
SVM_PaDEL+MACCS	0	86	0.55	0.10	0.53	0.55	0.59	0.51	0.52	0.57
SVM_PaDEL+MACCS	≥1	193	0.84	0.68	0.91	0.85	0.89	0.81	0.73	0.93
SVM_PaDEL+MACCS	≥2	147	0.90	0.80	0.96	0.92	0.96	0.88	0.79	0.98
SVM_PaDEL+MACCS	≥3	113	0.91	0.81	0.97	0.93	0.97	0.89	0.78	0.99
SVM_PaDEL+OASIS	0	86	0.53	0.07	0.52	0.54	0.56	0.51	0.51	0.56
SVM_PaDEL+OASIS	≥1	193	0.83	0.67	0.92	0.84	0.87	0.81	0.73	0.92
SVM_PaDEL+OASIS	≥2	147	0.9	0.79	0.96	0.91	0.96	0.87	0.77	0.98
SVM_PaDEL+OASIS	≥3	113	0.91	0.81	0.97	0.93	0.97	0.89	0.78	0.99

^1^ Number of consecutive nearest neighbors in the training data having the same activity class assigned as the one predicted for the test compounds. Abbreviations: ACC, accuracy; AUC, area under the receiver operating characteristic curve; CCR, correct classification rate; MCC, Matthews correlation coefficient; NPV, negative predictive value; PPV, positive predictive value; Se, sensitivity; Sp, specificity.

## Supplementary Materials

Supplementary Materials can be found at https://www.mdpi.com/1422-0067/20/19/4833/s1.

## Abbreviations

ACC	accuracy
ACD	allergic contact dermatitis
AUC	area under the receiver operating characteristic curve
CCR	correct classification rate
LLNA	local lymph node assay
MCC	Matthews correlation coefficient
MOE	Molecular Operating Environment
NERDD	New E-Resource for Drug Discovery
NPV	negative predictive value
PCA	principal component analysis
RBF	radial basis function
RF	random forest
PPV	positive predictive value
Se	sensitivity
Sp	specificity
SVM	support vector machine
