# Supplementary material for: Skin Doctor: Machine Learning Models for Skin Sensitization Prediction that Provide Estimates and Indicators of Prediction Reliability
Source: Int J Mol Sci. 2019 Sep 28;20(19):4833. doi: 10.3390/ijms20194833 (PMC6801714; doi:10.3390/ijms20194833)
Supplement: Supplementary file 1 [file ijms-20-04833-s001.pdf]

International Journal of
*Molecular Sciences*

MDPI

*Supporting Information for*

# Skin Doctor: Machine Learning Models for Skin Sensitization Prediction that Provide Estimates and Indicators of Prediction Reliability

**Anke Wilm [1,2], Conrad Stork [1], Christoph Bauer [3,4], Andreas Schepky [5], Jochen Kühnl [5] and Johannes Kirchmair [1,3,4*]**

1 Center for Bioinformatics, Universität Hamburg, Hamburg, Germany
2 HITeC e.V, Hamburg, Germany
3 Department of Chemistry, University of Bergen, Bergen, Norway
4 Computational Biology Unit (CBU), University of Bergen, Bergen, Norway
5 Front End Innovation, Beiersdorf AG, Hamburg, Germany

* Correspondence: kirchmair@zbh.uni-hamburg.de; Tel.: +49-40-42838-7303.

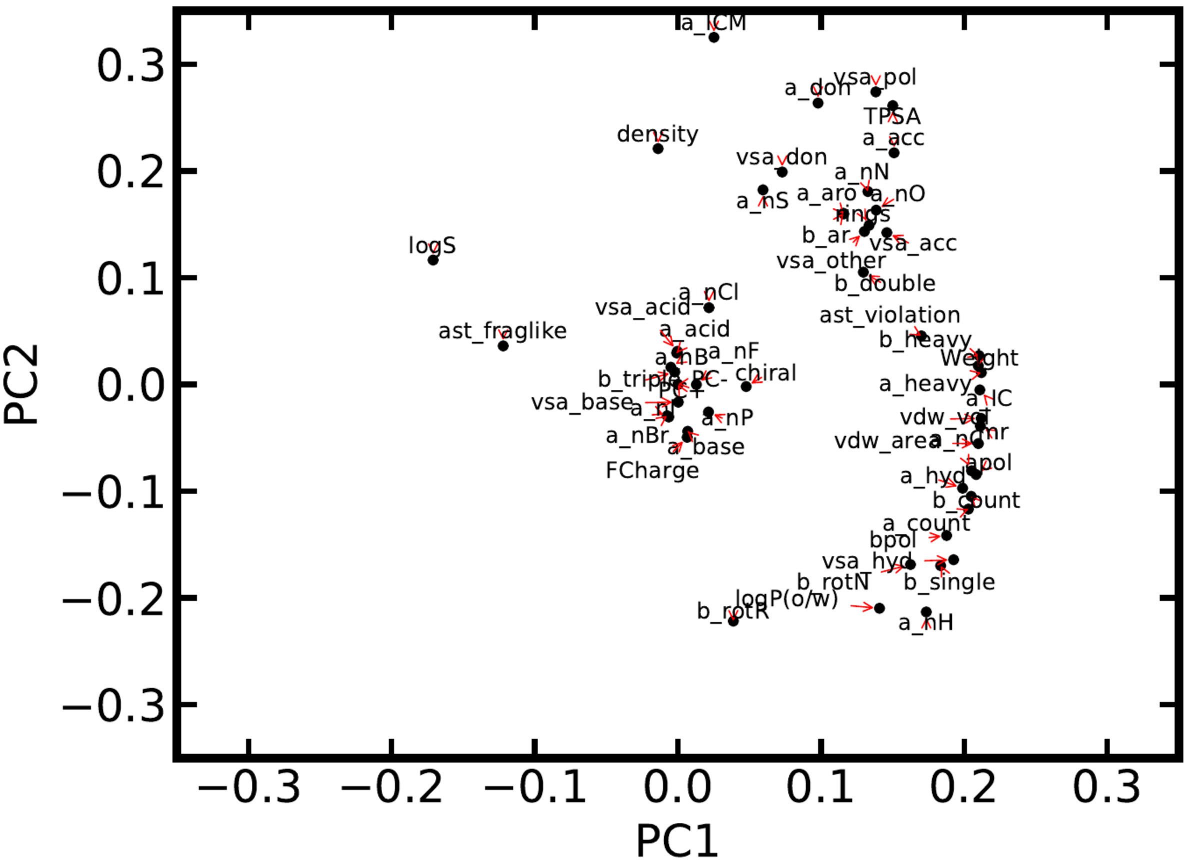


**Figure S1.** Enlarged version of the loadings plot from Figure 5B. For an explanation of the abbreviations see Table S1.

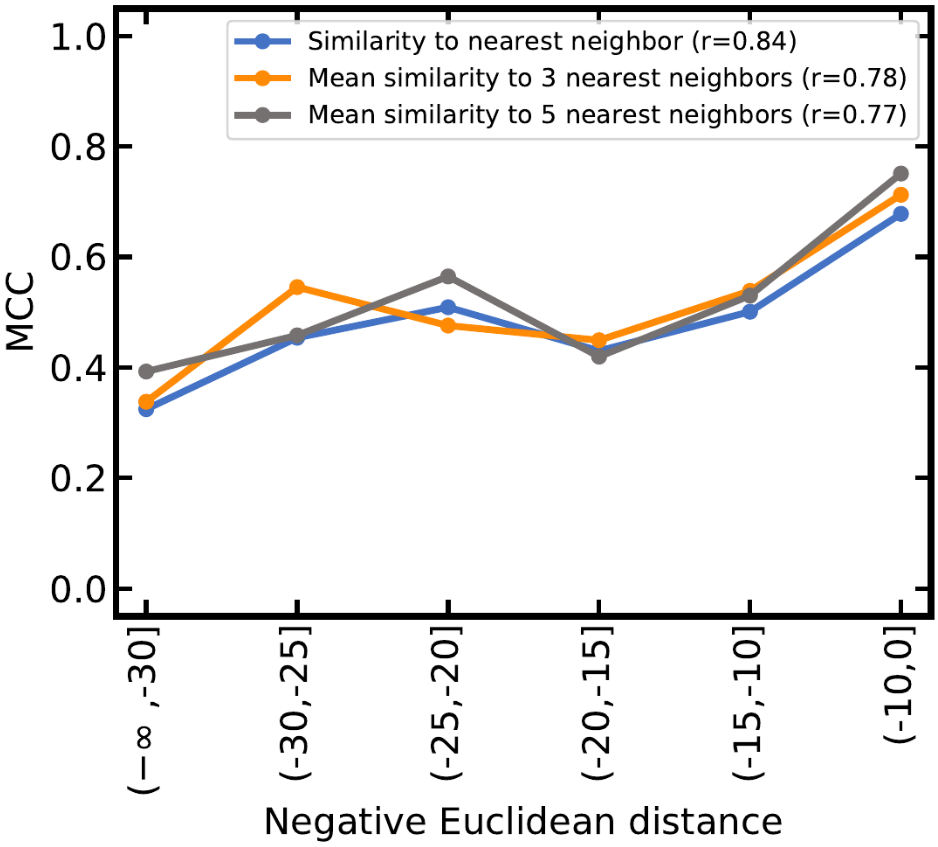


**Figure S2.** Correlation between molecular similarity measured as negative Euclidean distance in PaDEL space for the SVM_PaDEL model. Number of compounds in each bin are reported in Table S6.

**Table S1.** Descriptors Used for the PCA and Explanation of the Abbreviations.

| Descriptor | Explanation |
|---|---|
| apol | Polarizabilities of all atoms in molecule (as sum) |
| ast_fraglike | Binary Astex fragment-likeness |
| ast_violation | Number of Astex fragment-likeness violations |
| a_acc | H-bond acceptor atom count |
| a_acid | Acidic atom count |
| a_aro | Aromatic atom count |
| a_base | Basic atom count |
| a_count | Atom count |
| a_don | H-bond donor count |
| a_heavy | Heavy atom count |
| a_hyd | Hydrophobic atom count |
| a_IC | Total atom information content |
| a_ICM | Mean atom information content |
| a_nB | Boron atom count |
| a_nBr | Bromine atom count |
| a_nC | Carbon atom count |
| a_nCl | Chlorine atom count |
| a_nF | Fluorine atom count |
| a_nH | Hydrogen atom count |
| a_nI | Iodine atom count |
| a_nN | Nitrogen atom count |
| a_nO | Oxygen atom count |
| a_nP | Phosphorus atom count |
| a_nS | Sulfur atom count |
| bpol | Bonded atom polarizability difference |
| b_ar | Number of aromatic bonds |
| b_count | Number of bonds |
| b_double | Number of double bonds |

| | |
|---|---|
| b_heavy | Number of bonds between heavy atoms |
| b_rotN | Number of rotatable bonds |
| b_rotR | Fraction of rotatable bonds |
| b_single | Number of single bonds |
| b_triple | Number of triple bonds |
| chiral | Number of chiral centers |
| density | Molecular mass density |
| FCharge | Total charge of the molecule |
| logP(o/w) | Log of the octanol/water partition coefficient |
| logS | Log of the aqueous solubility (mol/L) |
| mr | Molecular refractivity |
| PC+ | Total positive partial charge |
| PC- | Total negative partial charge |
| rings | Number of rings |
| TPSA | Polar surface area (Å$^2$) |
| vdw_area | Area of van der Waals surface (Å$^2$) |
| vdw_vol | Van der Waals volume (Å$^3$) |
| vsa_acc | Approximation to the sum of VDW surface areas (Å$^2$) of pure hydrogen bond acceptors |
| vsa_acid | Approximation to the sum of VDW surface areas of acidic atoms (Å$^2$) |
| vsa_base | Approximation to the sum of VDW surface areas of basic atoms (Å$^2$) |
| vsa_don | Approximation to the sum of VDW surface areas of pure hydrogen bond donors |
| vsa_hyd | Approximation to the sum of VDW surface areas of hydrophobic atoms (Å$^2$) |
| vsa_other | Approximation to the sum of VDW surface areas (Å$^2$) of atoms typed as "other" |
| vsa_pol | Approximation to the sum of VDW surface areas (Å$^2$) of polar atoms |
| Weight | Molecular weight |

**Table S2.** Ten Most Prevalent Murcko Scaffolds in the LLNA Data Set.[1]

| | LLNA of Alves et al. | LLNA of Di et al. | Merged LLNA | Cosmetics | Drugs | Pesticides |
|---|---|---|---|---|---|---|
| 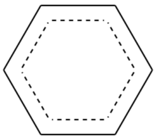 | 30.12% | 23.44% | 27.04% | 27.50% | 10.72% | 23.46% |
| 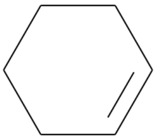 | 1.32% | 1.85% | 1.73% | 3.59% | 0.21% | |
| 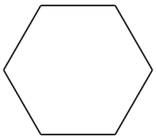 | 2.05% | 1.56% | 2.04% | 3.55% | 0.54% | 0.49% |
| 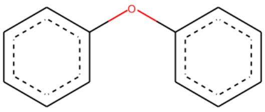 | 0.29% | 0.28% | 0.20% | 0.30% | 0.32% | 2.75% |
| 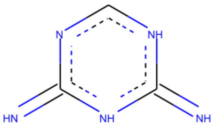 | 0.15% | 0.14% | 0.10% | 0.07% | | 1.94% |
| 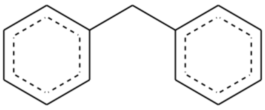 | 1.75% | 1.14% | 1.53% | 1.04% | 0.80% | 1.62% |
| 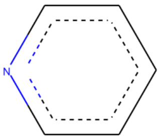 | 1.90% | 1.56% | 1.53% | 0.85% | 0.75% | 1.13% |
| 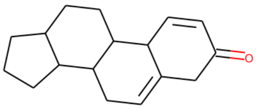 | 0.15% | 0.14% | 0.10% | 0.04% | 1.39% | |

| | | | | | | |
|---|---|---|---|---|---|---|
| 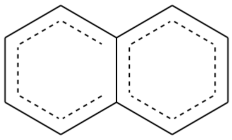 | 0.58% | 0.28% | 0.51% | 0.89% | 0.38% | 1.29% |
| 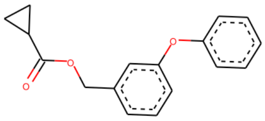 | | 0.14% | 0.10% | 0.07% | 0.05% | 1.29% |
| 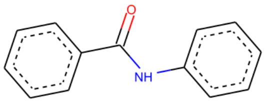 | 0.29% | 1.28% | 0.92% | 0.15% | 0.05% | 1.13% |
| 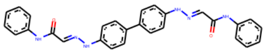 | 1.46% | 1.56% | 1.12% | 0.11% | | |
| 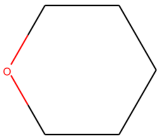 | 0.29% | 0.28% | 0.41% | 1.04% | 0.32% | |

[1] Reported are the percentages of compounds based on the indicated Murcko scaffolds among all compounds having a Murcko scaffold.

**Table S3.** Hyperparameters Selected During Grid Search.[1]

| | RF | | SVM | |
|---|---|---|---|---|
| **Name** | **n_estimators** | **max_features** | **C** | **gamma** |
| MOE2D | 250 | 0.4 | 1000 | 0.0001 |
| MOE2D53 | 250 | 0.4 | 1000 | 0.001 |
| Padel | 250 | 0.8 | 1 | 0.001 |
| MACCS | 1000 | sqrt | 1 | 0.1 |
| Morgan2 | 100 | 0.2 | 100 | 0.1 |
| OASIS | 10 | sqrt | 1 | 0.1 |
| Padel-Est | 1000 | 0.4 | 10 | 0.1 |
| Padel-Ext | 100 | 0.4 | 1 | 0.01 |
| MOE2D+Padel | 500 | None | 1 | 0.001 |
| MOE2D+MACCS | 500 | 0.2 | 10 | 0.01 |
| MOE2D+Morgan2 | 500 | 0.4 | 10 | 0.001 |
| MOE2D+OASIS | 100 | None | 100 | 0.001 |
| MOE2D+Padel-Est | 1000 | 0.4 | 10 | 0.01 |
| MOE2D+Padel-Ext | 1000 | sqrt | 10 | 0.001 |
| Padel+MACCS | 500 | 0.4 | 1 | 0.001 |
| Padel+Morgan2 | 1000 | 0.2 | 100 | 0.001 |
| Padel+OASIS | 500 | 0.6 | 1 | 0.001 |
| Padel+Padel-Est | 1000 | sqrt | 1 | 0.001 |
| Padel+Padel-Ext | 50 | 0.8 | 1 | 0.001 |
| MACCS+Morgan2 | 50 | 0.8 | 10 | 0.01 |
| MACCS+OASIS | 50 | None | 1 | 0.1 |
| MACCS+Padel-Est | 250 | sqrt | 1 | 0.1 |
| MACCS+Padel-Ext | 50 | 0.2 | 1 | 0.01 |
| Morgan2+OASIS | 100 | sqrt | 100 | 0.1 |
| Morgan2+Padel-Est | 250 | sqrt | 10 | 0.01 |
| Morgan2+Padel-Ext | 1000 | sqrt | 1 | 0.01 |
| OASIS+Padel-Est | 50 | 0.4 | 10 | 0.1 |
| OASIS+Padel-Ext | 1000 | 0.6 | 1 | 0.01 |
| Padel-Est+Padel-Ext | 250 | 0.6 | 1 | 0.01 |

[1] Definitions of the individual descriptor sets are provided in Table 2.

**Table S4.** Matthews Correlation Coefficients for the RF Models.[1]

| | MOE2D | PaDEL | Morgan2 | PaDEL-Ext | PaDEL-Est | MACCS | OASIS |
|---|---|---|---|---|---|---|---|
| **MOE2D** | 0.44 | 0.48 | 0.46 | 0.45 | 0.45 | 0.44 | 0.45 |
| **PaDEL** | | 0.48 | 0.49 | 0.49 | 0.47 | 0.49 | 0.49 |
| **Morgan2** | | | 0.46 | 0.44 | 0.48 | 0.44 | 0.44 |
| **PaDEL-Ext** | | | | 0.42 | 0.43 | 0.43 | 0.43 |
| **PaDEL-Est** | | | | | 0.43 | 0.46 | 0.48 |
| **MACCS** | | | | | | 0.47 | 0.47 |
| **OASIS** | | | | | | | 0.27 |

[1] The diagonal reports MCC values for models based on a single set of descriptors.

**Table S5.** Matthews Correlation Coefficients for the SVM Models.[1]

| | MOE2D | PaDEL | Morgan2 | PaDEL-Ext | PaDEL-Est | MACCS | OASIS |
|---|---|---|---|---|---|---|---|
| **MOE2D** | 0.48 | 0.5 | 0.5 | 0.5 | 0.5 | 0.5 | 0.55 |
| **PaDEL** | | 0.5 | 0.51 | 0.51 | 0.5 | 0.51 | 0.5 |
| **Morgan2** | | | 0.39 | 0.48 | 0.43 | 0.46 | 0.43 |
| **PaDEL-Ext** | | | | 0.47 | 0.47 | 0.46 | 0.47 |
| **PaDEL-Est** | | | | | 0.44 | 0.49 | 0.47 |
| **MACCS** | | | | | | 0.47 | 0.48 |
| **OASIS** | | | | | | | 0.29 |

[1]The diagonal reports MCC values for models based on a single set of descriptors.

**Table S6.** Number of Compounds with Specified negative Euclidean distance to 1, 3 and 5 Nearest Neighbors of SVM_PaDEL model in PaDEL space.

| | **(-∞ ,-30]** | **(-30,-25]** | **(-25,-20]** | **(-20,-15]** | **(-15,-10]** | **(-10,0]** |
|---|---|---|---|---|---|---|
| Similarity to nearest neighbor | 138 | 112 | 193 | 267 | 259 | 140 |
| Mean similarity to 3 nearest neighbors | 174 | 148 | 237 | 295 | 207 | 48 |
| mean similarity to 5 nearest neighbors | 200 | 174 | 259 | 288 | 171 | 17 |

International Journal of *Molecular Sciences*

MDPI

**Table S7.** Number of Compounds with Specified Mean Tanimoto Similarity to 1, 3 and 5 Nearest Neighbors.

| Model | Number of neighbors considered | Mean Tanimoto similarity | | | | | |
|---|---|---|---|---|---|---|---|
| | | **[0 ,0.5]** | **(0.5,0.6]** | **(0.6,0.7]** | **(0.7,0.8]** | **(0.8,0.9]** | **(0.9,1]** |
| SVM_MOE2D+OASIS | 1 | 24 | 89 | 218 | 327 | 244 | 226 |
| | 3 | 38 | 140 | 339 | 334 | 154 | 123 |
| | 5 | 53 | 207 | 374 | 289 | 140 | 65 |
| SVM_PaDEL+OASIS | 1 | 19 | 92 | 198 | 320 | 252 | 228 |
| | 3 | 34 | 134 | 317 | 343 | 164 | 117 |
| | 5 | 44 | 204 | 362 | 297 | 132 | 70 |
| SVM_PaDEL | 1 | 19 | 92 | 198 | 320 | 252 | 228 |
| | 3 | 34 | 134 | 317 | 343 | 164 | 117 |
| | 5 | 44 | 204 | 362 | 297 | 132 | 70 |
| RF_MACCS | 1 | 25 | 89 | 207 | 335 | 242 | 234 |
| | 3 | 38 | 138 | 329 | 344 | 168 | 115 |
| | 5 | 56 | 204 | 374 | 296 | 135 | 67 |
| SVM_PaDEL+MACCS | 1 | 19 | 92 | 198 | 320 | 252 | 228 |
| | 3 | 34 | 134 | 317 | 343 | 164 | 117 |
| | 5 | 44 | 204 | 362 | 297 | 132 | 70 |

**Table S8.** Number of Compounds with Specified Distances Between the Prediction Probability and the Decision Threshold.

| | Distance | | | | | | | |
|---|---|---|---|---|---|---|---|---|
| **Model** | **[0 ,0.25]** | **(0.25 - 0.5]** | **(0.5 - 0.75]** | **(0.75 - 1]** | **(1 - 1.25]** | **(1.25 - 1.5]** | **(1.5 - 1.75]** | **(1.75,∞)** |
| SVM_MOE2D+OASIS | 124 | 159 | 133 | 125 | 121 | 100 | 88 | 278 |
| SVM_PaDEL+OASIS | 183 | 233 | 198 | 174 | 173 | 91 | 48 | 9 |
| SVM_PaDEL | 180 | 238 | 193 | 177 | 172 | 92 | 47 | 10 |
| SVM_PaDEL+MACCS | 182 | 237 | 198 | 172 | 174 | 90 | 48 | 8 |
| | **[0 ,0.1]** | **(0.1,0.15]** | **(0.15,0.2]** | **(0.2,0.25]** | **(0.25,0.3]** | **(0.3,0.35]** | **(0.35,0.4]** | **(0.4,0.5)** |
| RF_MACCS | 237 | 134 | 126 | 128 | 126 | 113 | 73 | 195 |

**Table S9.** Number of Compounds with Specified Numbers of Consecutive Nearest Neighbors with Same Activity as Predicted.

| **Model** | **0** | **1** | **2** | **3** | **4** | **5 or more** |
|---|---|---|---|---|---|---|
| SVM_MOE2D+OASIS | 308 | 201 | 142 | 102 | 71 | 304 |
| SVM_PaDEL+OASIS | 295 | 213 | 124 | 94 | 83 | 300 |
| SVM_PaDELl | 295 | 213 | 124 | 94 | 83 | 300 |
| RF_MACCS | 329 | 187 | 135 | 104 | 78 | 299 |
| SVM_PaDEL+MACCS | 294 | 213 | 124 | 94 | 83 | 301 |
